# Double Transverse Mini‐Incision Repair for Proximal Achilles Tendon Tears

**DOI:** 10.1002/atn2.70202

**Published:** 2026-07-16

**Authors:** Taylor Stauffer, Ryann Davie, Josh Bram, Emily Smith, Tony Wanich

**Affiliations:** ^1^ Hospital for Special Surgery New York City New York U.S.A.

## Abstract

Recent literature showing equivalent outcomes for surgical and nonoperative management of Achilles tendon ruptures emphasizes the importance of proper indications for surgical repair. Various techniques for tendon repair via reapproximation of the torn ends have been described, such as open, mini‐open, and minimally invasive, each with their own advantages and disadvantages. This technique note describes a modified double transverse mini‐open repair procedure for proximal Achilles tendon tears that addresses the limited mobility of the distal stump seen with proximal tears, allowing for reapproximation and high healing rate. Advantages of this technique include simplicity, high reproducibility, and limited disruption of the posterior watershed region of the superficial skin and soft tissue overlying the tendon. This decreases the risk of wound complications that can lead to surgical site infections and skin‐tendon adhesion.

## Graphical abstract

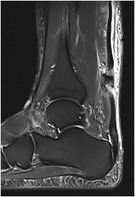

**VIDEO 1:** This video takes the viewer through the operative steps for a double transverse mini‐incision repair for proximal Achilles tendon tears. It shows photos and videos of the patient supine on a flat Jackson operating table with the left leg draped in sterile fashion. The foot hangs off the distal aspect of the bed. The incisions are marked and the viewer is taken through the approach to the tendon in addition to its repair using suture tape. Postoperative rehabilitation protocol is also discussed at the end. Video content can be viewed at https://doi.org/10.1002/atn2.70202.

The current literature comparing nonoperative versus operative treatment of acute Achilles tendon rupture is controversial regarding optimal treatment and rerupture rates. Although some studies report equivalent rerupture and postoperative function, others cite reduced rerupture rates and superior outcomes in surgical patients.^1^, ^2^ However, open surgical repair is associated with an increased risk of soft tissue complications, stiffness, and wound infection.^3^ As a result, noninvasive or mini‐open techniques have been developed to mitigate these complications, in addition to conferring shorter operative times and improved patient satisfaction.^3^, ^4^ Noninvasive techniques are not without complication, as sural nerve injury is the most commonly cited event, reaching as high as 14% in cadaveric studies.^5^ Despite this limitation, many studies assessing minimally invasive Achilles repair have reported low rates of temporary sural nerve injury in addition to excellent patient‐reported outcomes and satisfaction.^3^, ^4^, ^6^, ^7^, ^8^, ^9^

A multitude of noninvasive techniques currently exist, including minimally invasive approaches with endoscopy, percutaneous repair, or hybrid approaches that encompass both mini‐incision and percutaneous components. Examples of these noninvasive assistive devices include the Achillon (Integra Lifesciences), the Dresden instrument, or the Percutaneous Achilles Repair System (PARS, Arthrex, Naples, FL) which have showed good outcomes.^10^, ^11^, ^12^ Disadvantages of these systems are the necessity of additional instrumentation with a possible learning curve, increased operative cost, and continued risk of nerve injury.^12^ Modified, instrument‐free approaches have thus been introduced using a dual percutaneous and mini‐incision approach to avoid sural nerve injury.^9^, ^13^ In a 2022 retrospective study, Xu et al. described a double transverse mini‐incision repair technique for acute Achilles tears, including proximal and distal <2 cm transverse incisions that did not extend past the lateral border of the Achilles. In their technique, the proximal stump was repaired open via a traditional Bunnell configuration while the distal stump was repaired percutaneously. Among all 20 patients, they observed no sural nerve deficits in addition to significant increases in American Orthopaedic Foot & Ankle Society scores postoperatively.^9^ A 2024 study comparing this same technique with a traditional percutaneous repair in midsubstance tears reported no difference in rerupture or return to sport, but did show significantly decreased patient‐reported feelings of “tightness” and “foot numbness” among transverse mini‐incision patients.^14^

In this technique note, we describe a modified double transverse mini‐open procedure for proximal Achilles tendon tears that allows for excellent reapproximation and high healing rates (Video 1). Advantages of this technique include its simplicity, high reproducibility, and the fact that additional instrumentation is not required. The transverse incision also limits disruption of the posterior watershed region of the superficial skin and soft tissue over the tendon. This significantly decreases the risk of wound complications that lead to surgical site infections and skin‐tendon adhesion.

## SURGICAL TECHNIQUE

### Preoperative Considerations

**FIGURE 1:**
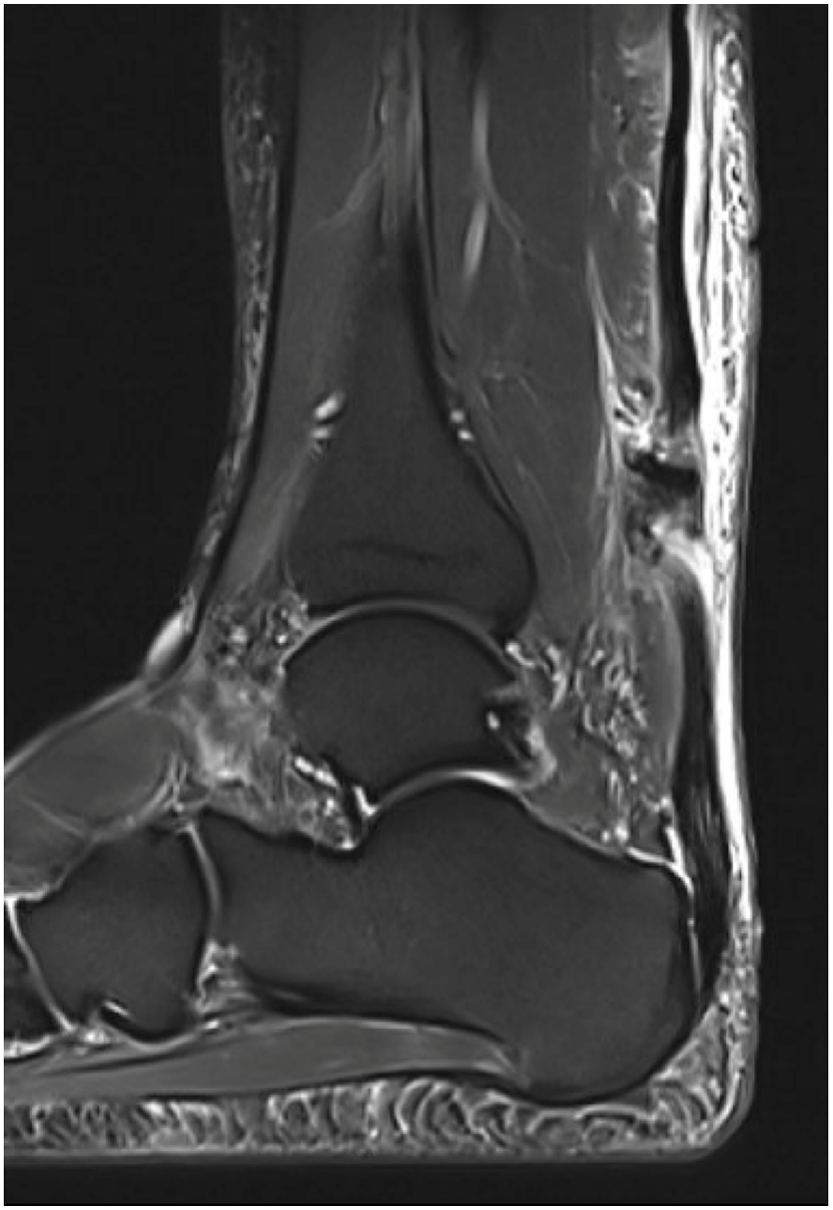
Preoperative supine T2‐weighted magnetic resonance imaging (MRI) of a left lower leg and foot showing a proximal Achilles tendon tear.

Preoperative assessment involves taking the patient's history and performing a physical examination to diagnose and evaluate an acute proximal Achilles tendon rupture. The senior author's preferred imaging modality is bedside ultrasound, which is a simple and inexpensive way to confirm the diagnosis.^15^ Other modalities include magnetic resonance imaging (Figure 1). Before positioning in the operating room, a neuraxial block and popliteal peripheral nerve block are performed by the anesthesia provider per the surgeon's preference. The patient is positioned prone on the operating table, and all bony prominences are well‐padded. The operative leg is then prepped and draped in a sterile fashion to the knee.

The toes are covered with Coban wrapping and the operative foot is allowed to drape off of the operating table.

### Surgical Approach to the Proximal Stump

With a marking pen, the location of the distal end of the proximal stump is marked. With a 15 blade, a 1 cm traverse incision is made approximately 1 cm proximal to this mark. This incision is carried down through skin and subcutaneous tissues using dissecting scissors to the level of the paratenon. Skin hooks are used to retract the skin for visualization. A transverse incision is made through the paratenon with a 15 blade and the proximal stump of the Achilles tendon is identified. At this point, Senn retractors or skin hooks are used to aid with visualization. The tendon tear is then visualized and debrided with a 15 blade or a tonsil before attaching the proximal stump to 2 Allis clamps to retract and externalize it from the incision. Alternatively, as shown in the video, an Allis or a tonsil clamp can be placed transversely along the tendon distally, maintaining a few millimeters of distal tendon. This helps to keep the tendon externalized and assist with stump preparation.

### Proximal Stump Suture Preparation

**FIGURE 2:**
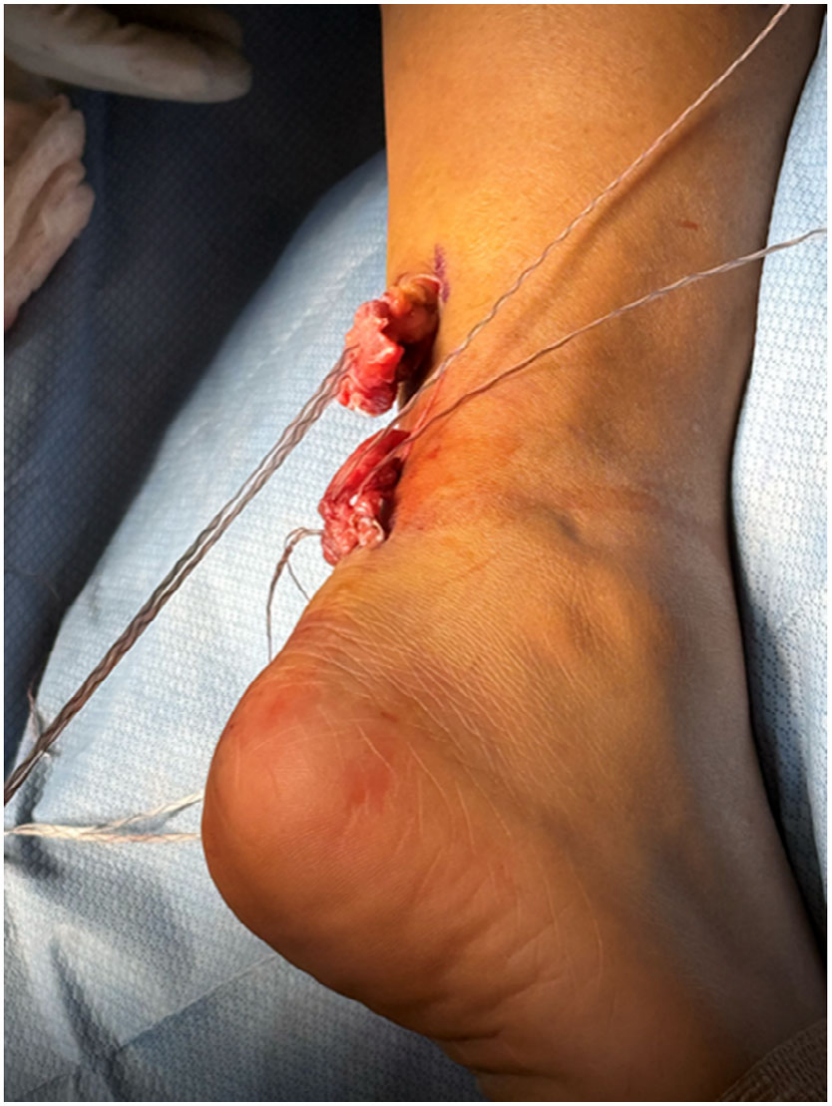
With the patient prone with the left foot hanging off the distal aspect of the operating table, the proximal and distal stumps are sutured using 1.3 mm SutureTape (Arthrex, Naples, FL) in a locking Kraków fashion at both the medial and lateral aspects of the tendon. These stumps will then be approximated to complete the repair once shuttled through the appropriate incisions.

Two 1.3 mm SutureTape (Arthrex, Naples, FL) are used to whipstitch the proximal tendon in a locking Krackow fashion along the medial and lateral aspects of the tendon, respectively. Each stitch is started at the distal aspect of the tendon, worked proximally with approximately 4 passes, and then brought back distally with the same number of passes in traditional Krackow fashion. Ultimately, there should be 4 SutureTape strands coming from the proximal stump, 2 on the medial and 2 on the lateral side of the tendon. These can be secured and kept organized with 2 clamps, respectively. The proximal tear is then unclamped and attention is then turned to the distal portion (Figure 2).

### Surgical Approach to the Distal Stump

The location of the proximal end of the distal stump is marked with a marking pen and a 1 cm traverse incision is made approximately 1 cm distal. Similarly to the first incision, this second incision is carried down to the level of the paratenon with the same instruments as mentioned above. A transverse incision is made through the paratenon and the distal stump of the Achilles tendon is identified. The tear is then visualized and debrided before attaching the distal stump to 2 Allis clamps to retract and externalize it from the incision.

### Distal Stump Suture Preparation

Similarly as mentioned above for the proximal stump, two 1.3 mm SutureTape (Arthrex, Naples, FL) are used to whipstitch the distal tendon in a locking Krackow fashion along the medial and lateral aspects of the tendon stump. Each stitch is started at the distal aspect of the tendon, worked proximally with approximately 4 passes, and then brought back distally with the same number of passes in traditional Krackow fashion. Ultimately, there should be 4 SutureTape strands coming from the proximal stump, 2 on the medial and 2 on the lateral side of the tendon. These can be secured and kept organized with 2 clamps, respectively. Once the sutures are passed, the distal stump is unclamped.

### Achilles Reapproximation

**FIGURE 3:**
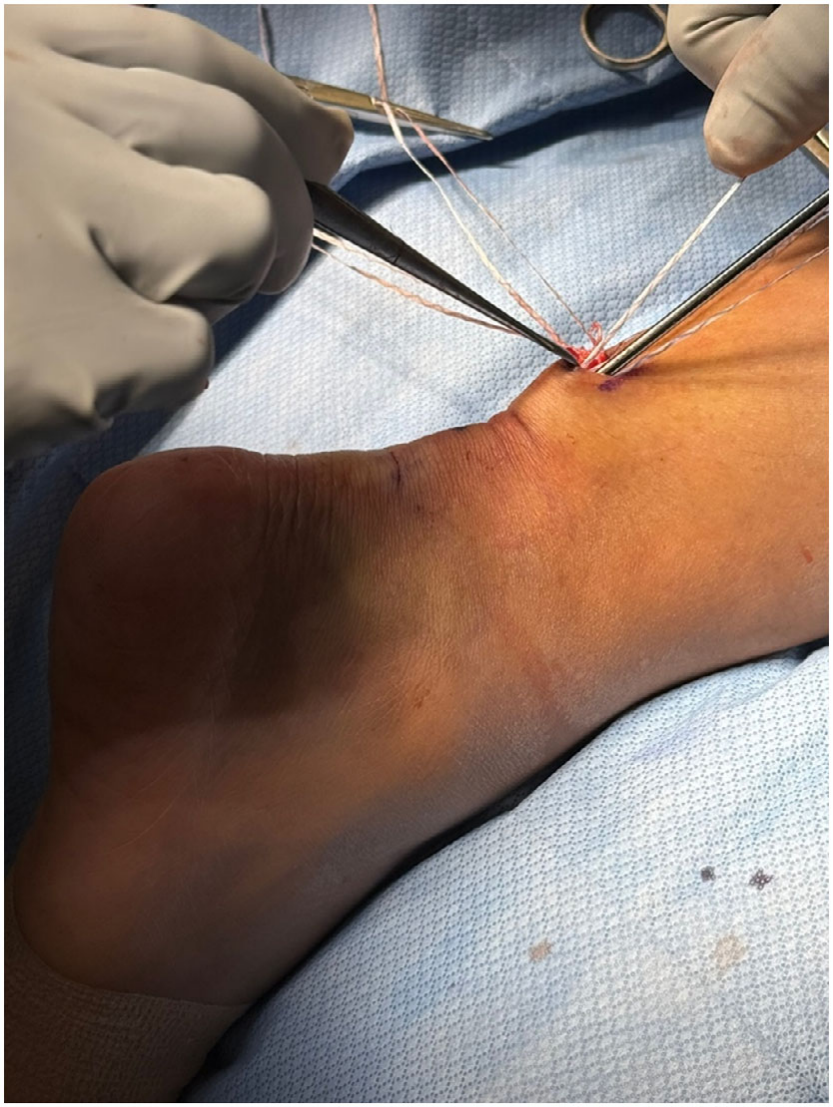
With the patient prone with the left foot hanging off the distal aspect of the operating table, the 2 tendon stumps are then brought into apposition by tying the sutures from the opposing ends together using an arthroscopic knot pusher (Arthrex, Naples, FL) through the proximal incision. This allows for anatomic reduction of the proximal and distal stumps with restoration of normal resting tension. This is done with the foot held in plantarflexion.

With a 15 blade, a few millimeters of distal end of the proximal stump and the proximal end of the distal stump are cut transversely to create fresh ends for reapproximation. A ruler may be used as a surface to cut on. Using an arthroscopic grasper passed from proximal to distal, the sutures from the distal stump are then shuttled through the paratenon sheath to the proximal incision, being sure to maintain the medial sided SutureTape on the medial aspect of the incision and the lateral sided SutureTape on the lateral aspect of the incision (Figure 3). At this point, there should be 4 pairs of SutureTape coming from the proximal aspect of the incision (2 pairs from the distal stump, 2 pairs from the proximal stump). The 2 tendon stumps are then brought into apposition by tying the sutures from the opposing ends together using an arthroscopic knot pusher (Arthrex, Naples, FL) through the proximal incision. A total of 4 knots are made, 2 on the lateral aspect of the tendons and 2 on the medial aspects of the tendon adjoining the accompanying SutureTape from the opposing stump. The excess suture is cut with Mayo scissors. This allows for anatomic reduction of the proximal and distal stumps with restoration of normal resting tension. The repair tension is then checked with confirmation of normalization of the Thompson test with the knee bent at 90°.

### Wound Closure

Both incisions are thoroughly irrigated with normal saline solution and the paratenon is reapproximated using 3‐0 Vicryl sutures. The deep dermis is closed used 2‐0 Vicryl sutures followed by a running Monocryl for the subcuticular layer. Steri‐strips and dry sterile dressings are applied. The patient is then placed in splint with the foot maintained in equinas.

### Postoperative Rehabilitation

The patient is seen 2 weeks postoperatively for a wound assessment. At this point, they are transitioned into a boot with wedges to maintain equinas. The patient is kept nonweightbearing for a total of 6 weeks. At 4 weeks, the patient begins physical therapy to regain baseline function, strength, and range of motion. Pearls and Pitfalls for the technique are listed in Table 1.

**TABLE 1:** Surgeon's Pearls and Pitfalls

Pearls	Pitfalls
‐ Passing beneath the intact paratenon and debriding ends to healthy tissue provides adequate tension for repair. ‐ Take the time to appropriate place incisions to ensure adequate tendon access.	‐ Knots are not directly visualized with arthroscopic knot tying; one must be facile with/practice this.

## DISCUSSION

Recent literature showing equivalent outcomes for surgical and nonoperative management of Achilles tendon ruptures emphasizes the importance of proper indications for surgical repair.^1^, ^2^ Various techniques for tendon repair via reapproximation of the torn ends have been described, such as open, mini‐open, and minimally invasive. Open and mini‐open repairs present the advantage of direct visualization, which provides for a theoretically more robust repair via the Krackow or Bunnell technique. Open repair also results in a smaller incidence of sural nerve injuries.^4^ A disadvantage of the open technique is the complication of surgical site infections, which comes with significant morbidity and cost for the patient. Also, open repair comes with potentially longer operative time compared with minimally invasive surgical repair.^3^ Minimally invasive techniques via percutaneous devices were designed to minimize serious soft‐tissue complications that came with open repair. However, with less visualization, minimally invasive techniques have showed a high risk of sural nerve injury and a higher risk of rerupture.^7^, ^8^, ^12^ Delayed healing may lead to failure and tendon elongation without satisfactory reapproximation of the tendon stumps.

Proximal Achilles tendon tears, within 5 cm of the musculotendinous junction of the gastrocnemius and soleus, present unique challenges with single incision mini‐open techniques due to lack of excursion of the distal stump. This mini dual incision technique has been developed to provide a simple and reproducible method with the combined advantages of open and percutaneous repair. Mini‐incisions proximal and distal, not directly superficial to the tendon repair site, further mitigate the risk of postoperative soft tissue infections. Direct visualization with mini‐incisions also decreases the risk of sural nerve injury and skin‐tendon adhesions. Compared with the percutaneous technique, the mini dual incision is limited by disruption of blood supply during approach and exposure of the paratenon.^6^, ^9^, ^14^ Transverse incisions perpendicular to its path may also put the sural nerve at increased risk of injury. However, the author believes that the risk of sural nerve injury is minimal with careful dissection and awareness of its relationship to the lateral tendon. Advantages and Disadvantages of this technique are described in Table 2.

**TABLE 2:** Advantages and Disadvantages

Advantages	Disadvantages
(+) Decreased risk of sural nerve injury	(−) Potential for less robust repair
(+) Additional instrumentation not needed, no additional operative cost	(−) Limited access
(+) Minimal learning curve	(−) Continued risk of nerve injury

The author's preferred technique for primary Achilles tendon repair for proximal tears includes an open dual mini‐incision with SutureTape (Arthrex, Naples, FL). This technique provides high‐strength suture fixation with reduced soft tissue dissection and limited contact between the repair wound closure, mitigating the risk of wound complications and sural nerve injury. This method addresses the limited mobility of the distal stump seen with proximal tears, allowing for reapproximation and high healing rates.

## DISCLOSURES

The authors (T.S., R.D., J.B., E.S., T.W.) declare that they have no known competing financial interests or personal relationships that could have appeared to influence the work reported in this article.
